# Propranolol sensitizes prostate cancer cells to glucose metabolism inhibition and prevents cancer progression

**DOI:** 10.1038/s41598-018-25340-9

**Published:** 2018-05-04

**Authors:** Laura Brohée, Olivier Peulen, Betty Nusgens, Vincent Castronovo, Marc Thiry, Alain C. Colige, Christophe F. Deroanne

**Affiliations:** 1 Laboratory of Connective Tissues Biology, GIGA-Cancer, University of Liège, 4000 Liège, Belgium; 2 Metastasis Research Laboratory, GIGA-Cancer, University of Liège, 4000 Liège, Belgium; 3 Laboratory of Cell Biology, GIGA-R, University of Liège, 20 rue des Pitteurs, 4020 Liège, Belgium

## Abstract

Propranolol, a widely used non-selective beta-adrenergic receptor blocker, was recently shown to display anticancer properties. Its potential to synergize with certain drugs has been also outlined. However, it is necessary to take into account all the properties of propranolol to select a drug that could be efficiently combined with. Propranolol was reported to block the late phase of autophagy. Hence, we hypothesized that in condition enhancing autophagy flux, cancer cells should be especially sensitive to propranolol. 2DG, a glycolysis inhibitor, is an anti-tumor agent having limited effect in monotherapy notably due to induction of pro-survival autophagy. Here, we report that treatment of cancer cells with propranolol in combination with the glycolysis inhibitor 2DG induced a massive accumulation of autophagosome due to autophagy blockade. The propranolol +2DG treatment efficiently prevents prostate cancer cell proliferation, induces cell apoptosis, alters mitochondrial morphology, inhibits mitochondrial bioenergetics and aggravates ER stress *in vitro* and also suppresses tumor growth *in vivo*. Our study underlines for the first time the interest to take advantage of the ability of propranolol to inhibit autophagy to design new anti-cancer therapies.

## Introduction

Cancer cells differ from normal cells by various hallmarks^1^ such as metabolism reprogramming that furnishes the energy and the (macro-)molecules allowing their rapid proliferation. Various metabolic pathways, including glycolysis and the biosynthesis of nucleic acid, protein or lipid, are altered in tumor cells and represent promising targets for anti-cancer therapies^2^. In a previous study, we highlighted the role of lipin-1, an enzyme participating to lipid anabolism, in regulating cancer cell phenotype and metabolism^3^. Lipins can regulate various cellular processes including gene expression, proliferation, migration and autophagy^4^. Through their phosphatidate phosphatase (PAP) activity, generating diacylglycerol (DAG) from phosphatidic acid (PA), they contribute to the fusion of autophagosomes with lysosomes and to autophagy clearance^5^. Autophagy is a ubiquitous catabolic process essential for cell survival under stress conditions^6,7^. Its role in cancer is complex and depends on biological factors, such as the tumor type or the driving oncogene^8^. It is also part of the pro-survival response of cancer cells to anti-cancer treatment and its inhibition was reported to enhance cancer cell death^9,10^.

Propranolol is a β-adrenergic receptor blocker notably used for the treatment of hypertension, myocardial infarction, anxiety and tremor^11^. Numerous studies have also demonstrated its efficacy for the treatment of infantile haemangiomas in human^12,13^. Propranolol is also an inhibitor of the PAP activity of lipins, which probably explains why it inhibits autophagy flux^3,14^. Furthermore, retrospective analyses reported a decreased risk of head and neck, stomach, colon and prostate cancers in patients receiving propranolol^15^.

2-deoxy-d-glucose (2DG), a glucose analog, is blocking the first critical step of glycolysis, therefore mimicking glucose deprivation, and leading to metabolic stress^16,17^. It interferes also with N-glycosylation of proteins thus preventing their normal folding and inducing endoplasmic reticulum (ER) stress^18,19^. Although 2DG is an anti-tumor agent, its effects in monotherapy are quite limited and combination with other drugs is required to achieve an efficient anticancer treatment^20^. Molecules aiming at inhibiting the 2DG-induced autophagy are particularly harmful to cancer cells^21,22^.

In view of their individual specific effects on cancer cells, we hypothesized that the combination of propranolol and 2DG might exert a synergistic toxic effect on cancer cells. Prostate cancer cells were used as an informative model in this study since this type of cancer is associated with an upregulation of lipogenic enzymes^23^, including lipin-1^3^, and with an elevated basal autophagy that protects prostate cancer cells from cytotoxic treatments^7^. Here, we report that combined treatment is effective on preventing prostate cancer cell proliferation, inducing cell apoptosis, altering mitochondrial morphology, inhibiting mitochondrial bioenergetics, aggravating ER stress *in vitro* and, most importantly, in suppressing tumor growth *in vivo*.

## Results

### Culture in low glucose or glycolysis inhibition sensitizes cancer cells to propranolol

**Figure 1:**
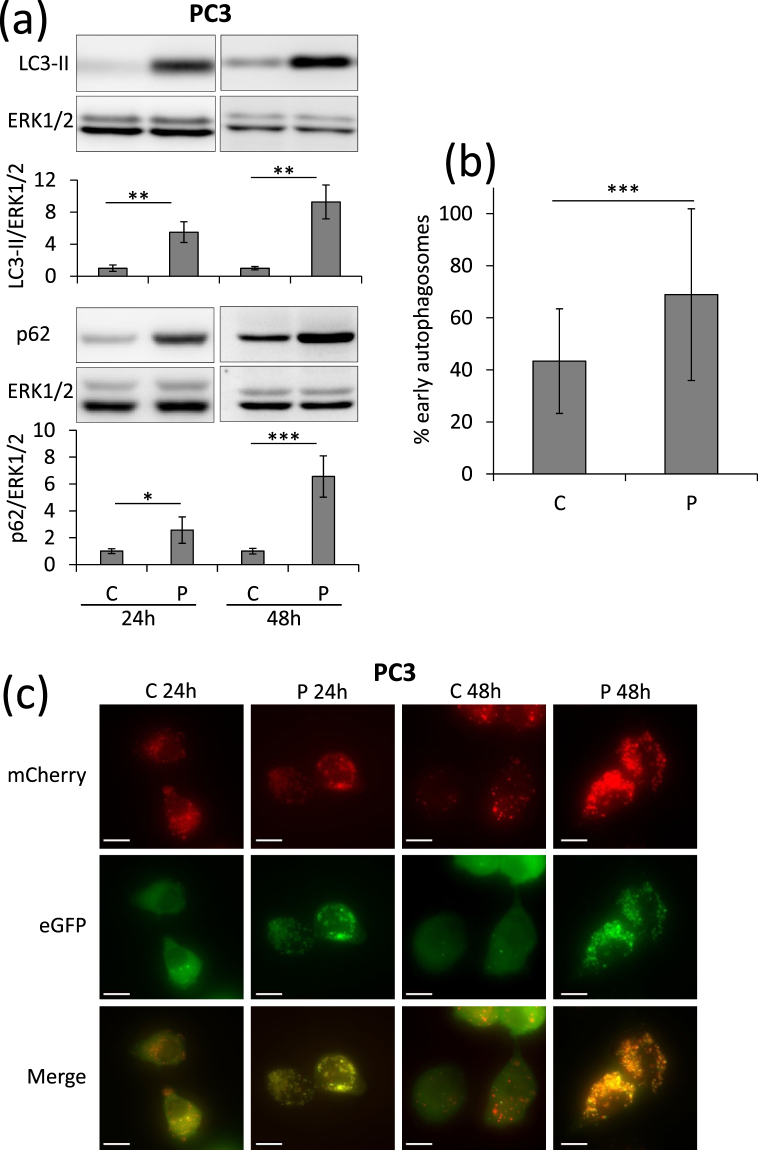
Propranolol blocks autophagy in PC3 cells and induces a massive accumulation of autophagosomes. PC3 cells were untreated (C) or treated with 100 µM propranolol (P) for 24 h or 48 h. (**a**) As compared to control (C), P treatment induces an increase of LC3-II and p62 in PC3 cells both at 24 and 48 h. Western blot quantifications were normalized on Erk1/2, used as control for protein loading. Results are expressed as fold increase compared to the control condition. (**b**,**c**) Autophagy flux was investigated by the transient overexpression of a LC3-eGFP-mCherry construct combined, or not, with P treatment (100 µM) for 24 or 48 hours. (**b**) Graphical representation of the percentages of early/late autophagosomes, after 48 h of treatment, as determined in at least 24 cells per condition (mean ± s.d.). Representative fluorescent microscopy photographs of each condition are shown in (**c**) (scale bars = 10 µm).

**Figure 2:**
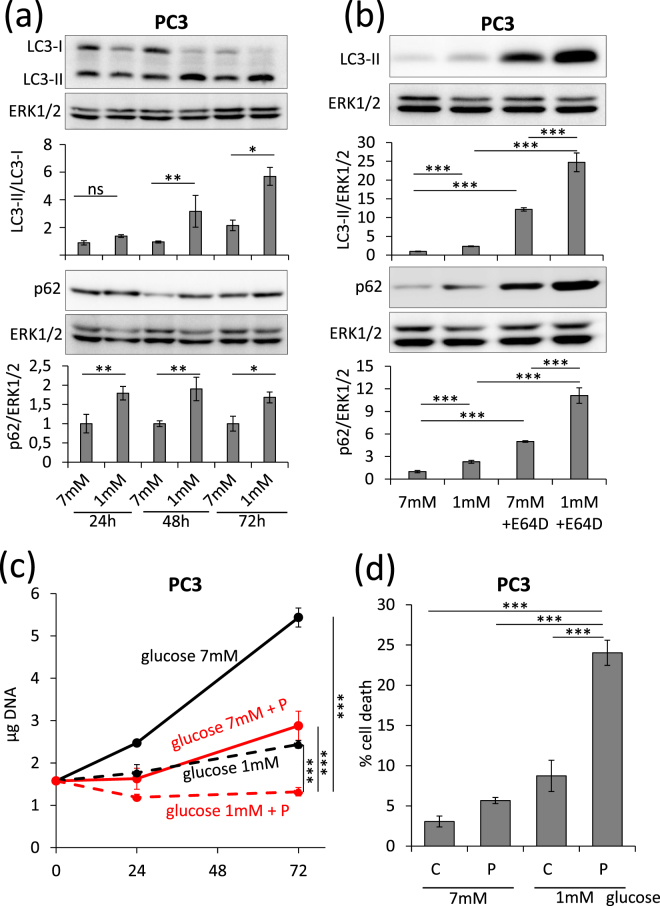
Low glucose condition increases autophagy and enhances sensitivity to propranolol in PC3 cells. PC3 cells were cultured in medium containing 7% dialyzed FBS and 1 mM or 7 mM glucose for the indicated times. (**a**) Autophagy was investigated by LC3-II/LC3-I and p62 western blotting followed by a normalization on Erk1/2 to control protein loading. Under low glucose PC3 cells have an increased autophagy flux. (**b**) Cells were challenged or not with 10 μg/ml of E64d, a cathepsin inhibitor, for 72 h. Treatment with E64d further enhances the low glucose-dependent accumulation of LC3-II and p62. (**c,d**) Cells were treated or not with 100 μM propranolol (P) for the indicated amount of time. (**c**) The proliferation of treated PC3 cells was measured as described in the “Materials and Methods”. P strongly inhibits PC3 cells proliferation in low glucose condition. (**d**) The percentage of death of PC3 cells was quantified by FACS after culture during 72 h. Propranolol induces cell death more efficiently in low than high glucose conditions. FACS analysis was performed after labeling PC3 cells with FITC-annexin V and propidium iodide. 10 000 events were collected for each experiment.

**Figure 3:**
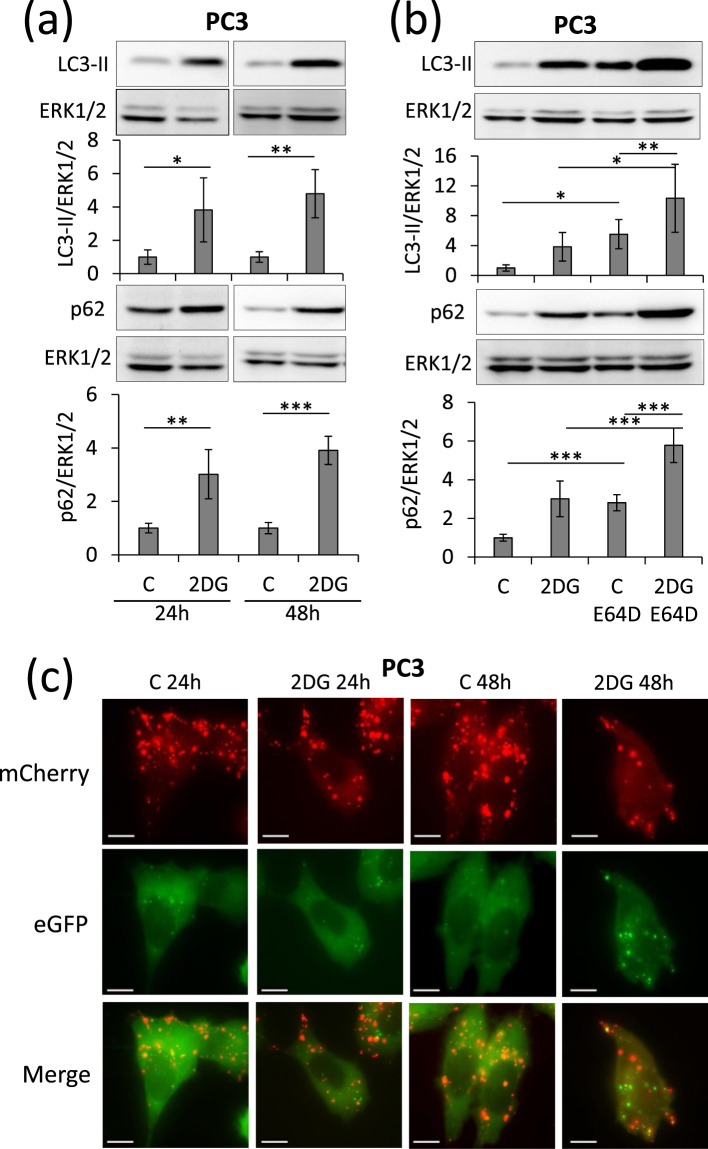
Glycolysis blockade by 2DG induced an increase of autophagy flux in PC3 cells. PC3 cells were treated or not with 10 mM 2DG for 24 or 48 hours. (**a**) 2DG treatment induces an increase of autophagy as showed by LC3-II and p62 accumulation both at 24 and 48 hours. (**b**) Cells were treated or not with 2DG and with (+E64d), or without, 10 μg/ml of the cathepsin inhibitor E64d for 48 h. Blocking the lysosomal proteases with E64d further enhances the 2DG-dependent accumulation of LC3-II and p62. Erk1/2 was used as protein loading control. (**c**) Autophagy flux was investigated by the transient overexpression of a LC3-eGFP-mCherry construct combined, or not, with 10 mM 2DG treatment for 24 or 48 h. Representative fluorescent microscopy photographs are shown (scale bars = 10 µm). No accumulation of autophagosomes is observed in 2DG condition.

**Figure 4:**
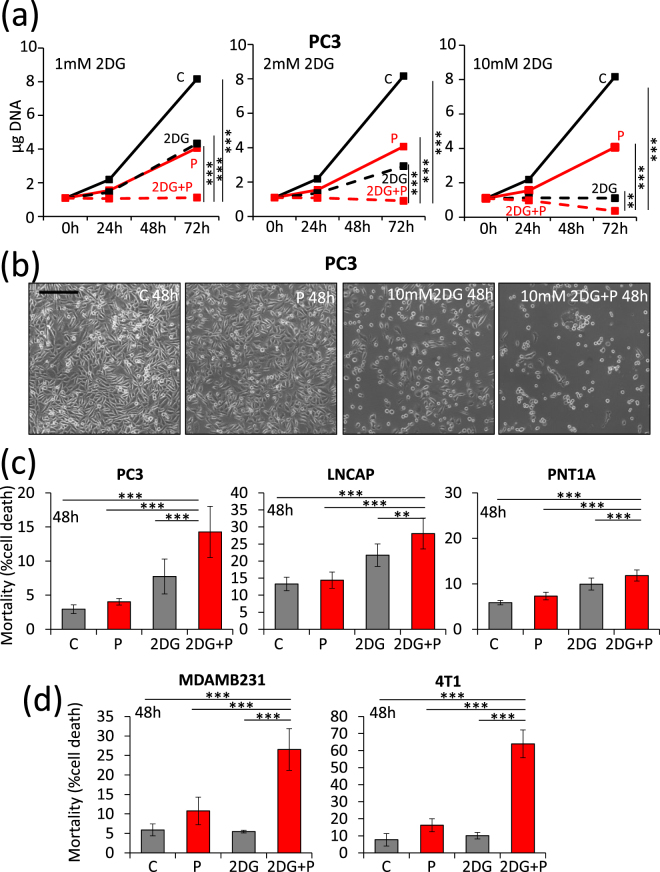
Propranolol and 2DG synergize in inducing arrest of proliferation and cell death of cancer cells. PC3 cells were treated with increasing concentrations of 2DG (1, 2 and 10 mM), propranolol (P) (100 µM) or both. (**a**) The proliferation of cells was measured by DNA content quantification as indicated in “Materials and Methods”. 2DG and P synergize to arrest PC3 cells. (**b**) Phase contrast microscopy photographs show the status of PC3 cells after 48 h of treatments (scale bar = 250 µm). (**c,d**) Cell death was measured by FACS analysis after labeling cells with FITC-annexin-V and propidium iodide. 10 000 events were collected for each experiment. (**c**) PC3, LnCaP and PNT1A were treated with 2DG (10 mM), propranolol (P) (100 µM) or both for 48 h. (**d**) MDAMB231 and 4T1 cells were treated with 2DG (10 mM and 2 mM respectively), propranolol (P) (100 µM and 50 µM respectively) or both for 48 h. Propranolol and 2DG synergize in inducing cancer cell death.

PC3 Prostate cancer cells have a basal autophagy flux essential for their tumorigenic phenotype^24^. When treated with propranolol, PC3 cells accumulate high amounts of the autophagy markers p62 and LC3-II (Fig. 1a). The use of cells expressing a fluorescent recombinant tagged LC3 (LC3-eGFP-mCherry) demonstrated that propranolol blocks autophagy clearance which results in a high percentage of early autophagosomes (Fig. 1b,c). LC3-II accumulates also in low glucose condition and this accumulation increases further upon addition of E64d, a cathepsin inhibitor (Fig. 2a,b). This suggested an increased autophagy flux which was further confirmed by recombinant tagged LC3 experiments demonstrating the lack of blockade of autophagy clearance (Supplementary Fig. S1). Since autophagy is an important mechanism allowing cells to survive to various stresses, including starvation, we hypothesized that the combination of low glucose and propranolol could be harmful for cells due to the blockade of autophagy. While treatment with low glucose or propranolol alone already inhibited the proliferation of PC3 cells, the combination of both completely blocked proliferation and even led to cell death since the cell number after 3 days of treatment, as evaluated by DNA content measurements, was lower than the originally seeded cell number (Fig. 2c). This was confirmed by direct evaluation of cell death. At 7 mM glucose propranolol increased cell death very slightly (+2.6%). By sharp contrast the effect of propranolol was markedly increased (+15.3%) at low glucose concentration (Fig. 2d). Glucose deprivation can be mimicked *in vitro* by culturing cells with 2DG, a glycolysis inhibitor. Accumulation of LC3-II was observed in the presence of 2DG and this accumulation was increased in the presence of E64d, suggesting an increased autophagy flux as observed with low glucose (Fig. 3a,b). Furthermore, the use of cells expressing a recombinant tagged LC3 demonstrated the lack of blockade of autophagy clearance in 2DG treated cells since no accumulation of early autophagosome was noticed (Fig. 3c and Supplementary Fig. S2). Based on these data, we hypothesized that propranolol might sensitize cancer cells to 2DG. While the proliferation of PC3 cells was significantly decreased in presence of propranolol (100 µM) or 2DG (1, 2 or 10 mM) alone (Fig. 4a), the combined treatment completely blocked the proliferation of PC3 cells (Fig. 4a) at 1 and 2 mM 2DG (p values < 0.001) and even led to a decreased cell number at 10 mM 2DG (p value < 0.01) after 3 days of culture as already observed for cultures at low glucose (Fig. 2c). These effects are clearly visible by phase contrast microscopy already at 48 h (Fig. 4b). While the cell shape and number were affected by propranolol or 2DG used alone, the combined treatment was much more potent. Cells were rounded, barely attached and their number was reduced as compared to respectively propranolol or 2DG used alone. Quantification of cell death by FACS confirmed the visual observations (Fig. 4c). The 2DG + P combination induces a 4.9 fold increase of cell death in the aggressive prostate cancer cells PC3. To broaden the significance of our results, we tested the effect of 2DG and propranolol alone or in combination on cells originating from another type of cancer. We observed that this effect was not limited to PC3 and prostate cancer cells since it induces also cell death in breast cancer cells (x4.5 and x8.3, as compared to controls, for MDAMB231 and 4T1 respectively) (Fig. 4d). Interestingly, the combination of both drugs had a less pronounced effect (about 2 fold increase) on two low- and non-tumorigenic prostatic cell lines (LnCaP and PNT1A) (Fig. 4c).

### The combination of 2DG and propranolol enhances autophagy blockade and exacerbates ER stress

**Figure 5:**
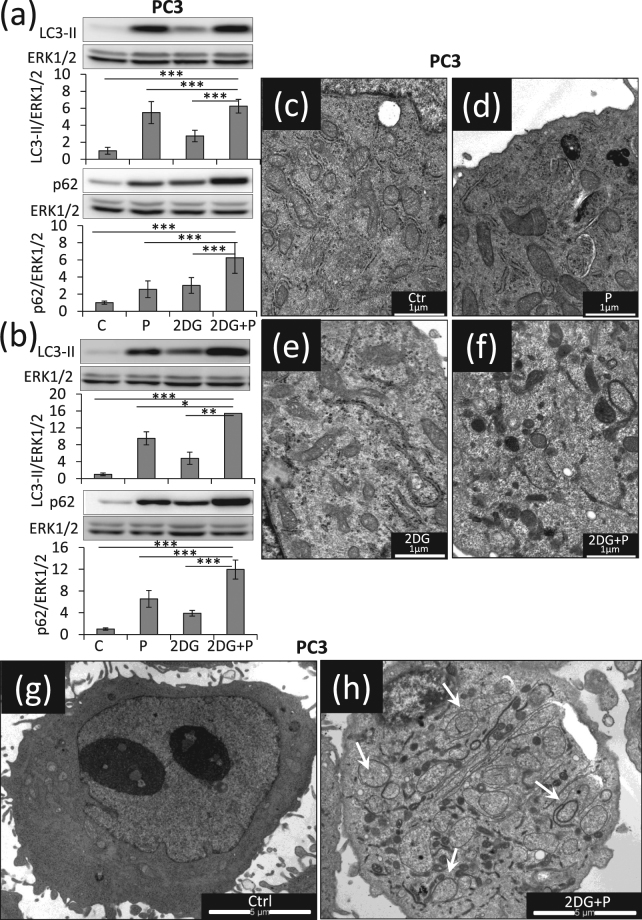
Propranolol and 2DG combined treatment results in massive accumulation of autophagosomes. PC3 cells were treated with 2DG (10 mM), propranolol (P) (100 µM), or both together, and were collected at the indicated times. (**a,b**) The levels of LC3-II and p62 were investigated by immune-blotting after 24 h or 48 h of treatment and normalized to Erk1/2 used as protein loading control. LC3-II and p62 further accumulate when the two drugs were added simultaneously, highlighting a dramatic accumulation of autophagosomes. Results are expressed as fold increase as compared to the control condition. (**c**–**h**) PC3 cells were collected after 24 h of treatments and prepared for electron microscopy analysis as detailed in “Materials and Methods”. Representative images of a cell in control condition (**c,g**), after exposure to P (**d**), to 2DG (**e**) or to the combined treatment (**f,h**) show an accumulation of autophagosomes in 2DG + P condition (see white arrows, **c** to **f** scale bar = 1 µm, (**g**,**h**) scale bars = 5 µm).

**Figure 6:**
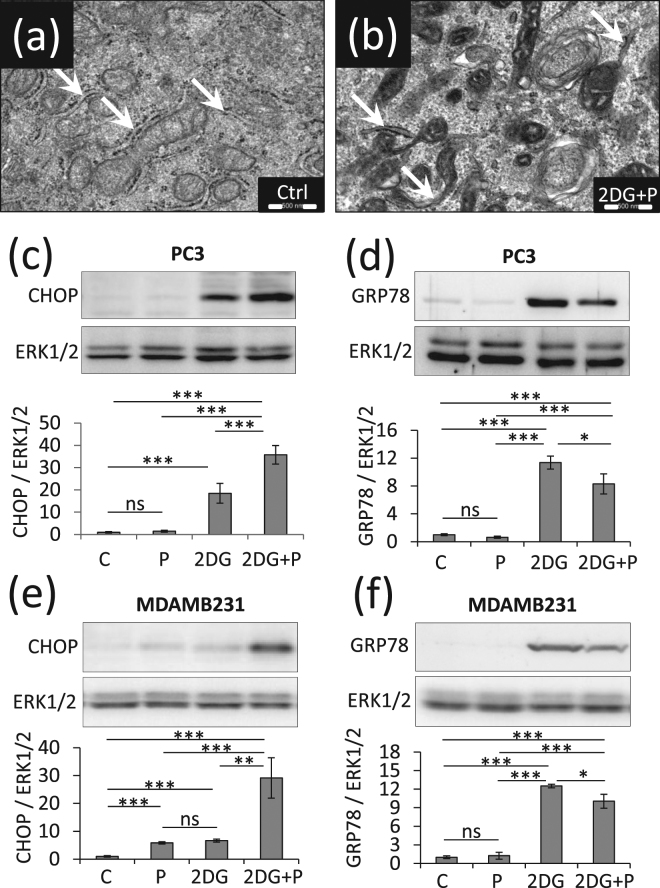
2DG + P induces ER stress in PC3 and MDAMB231 cells. PC3 cells and MDAMB231 were left untreated (C) or treated with 2DG (10 mM), propranolol (P) (100 µM) or both together (P+2DG) for the indicated times. (**a**,**b**) Electron microscopy comparison of control PC3 vs PC3 treated with 2DG + P (scale bars = 500 nm). The arrows pinpoint the ER structures. In 2DG+P condition ER appears condensed. PC3 (**c**,**d**) or MDAMB231 (**e**,**f**) cells were investigated for ER stress markers CHOP (**c**,**e**) and GRP78 (**d**,**f**). Signal quantifications are normalized to Erk1/2 as a control of protein loading. CHOP is drastically increased in cells treated with 2DG+P.

Since we hypothesized that the combination of 2DG and propranolol could impair autophagy, we evaluated the expression levels of LC3-II and p62. As compared to controls, propranolol or 2DG treatments led to the accumulation of LC3-II (x5.7 and x2.7, respectively) and p62 (x2.6 and 3.0, respectively) already after 24 hours. This effect was enhanced when the two drugs were added together (x6.5 for LC3II and x6.3 for p62) (Fig. 5a) and was further increased after 48 h (x15.4 for LC3II and of x11.9 for p62) (Fig. 5b). Similar results were also obtained with MDAMB231 cells (Supplementary Fig. S3). These data suggested a dramatic accumulation of autophagosomes due to a lack of maturation. This was confirmed by electron microscopy analyses which reveal a massive accumulation of vesicles formed by a double membrane containing cytoplasmic material (compare Fig. 5c,f–h). These analyses revealed also that PC3 cells exposed to the combined treatment are characterized by a denser ER lumen (see arrows Fig. 6a,b) and vesicle accumulation on the Golgi (Supplementary Fig. S4). In order to investigate the ER stress response, we measured the expression level of the pro-survival component of this system, glucose-related protein of molecular mass 78 (GRP78), and of its pro-apoptotic counterpart, C/EBP homologous protein (CHOP). Propranolol treatment did not induce GRP78 or CHOP expression while a significant increase was observed upon 2DG treatment (x15.6 for CHOP, x11.4 for GRP78), but without significantly changing the ratio between the two proteins. By contrast, the double treatment further enhanced CHOP expression (x35.7) but decreases the expression of GRP78 (x 8.3) as compared to 2DG treatment alone (x11.4) which strongly affects the CHOP/GRP78 ratio and suggest a dramatic aggravation of ER stress (Fig. 6c,d). A similar regulation of CHOP and GRP78 was also observed with MDAMB231 cells (Fig. 6e,f).

### Combining 2DG to propranolol leads to a strong inhibition of mitochondrial bioenergetics

**Figure 7:**
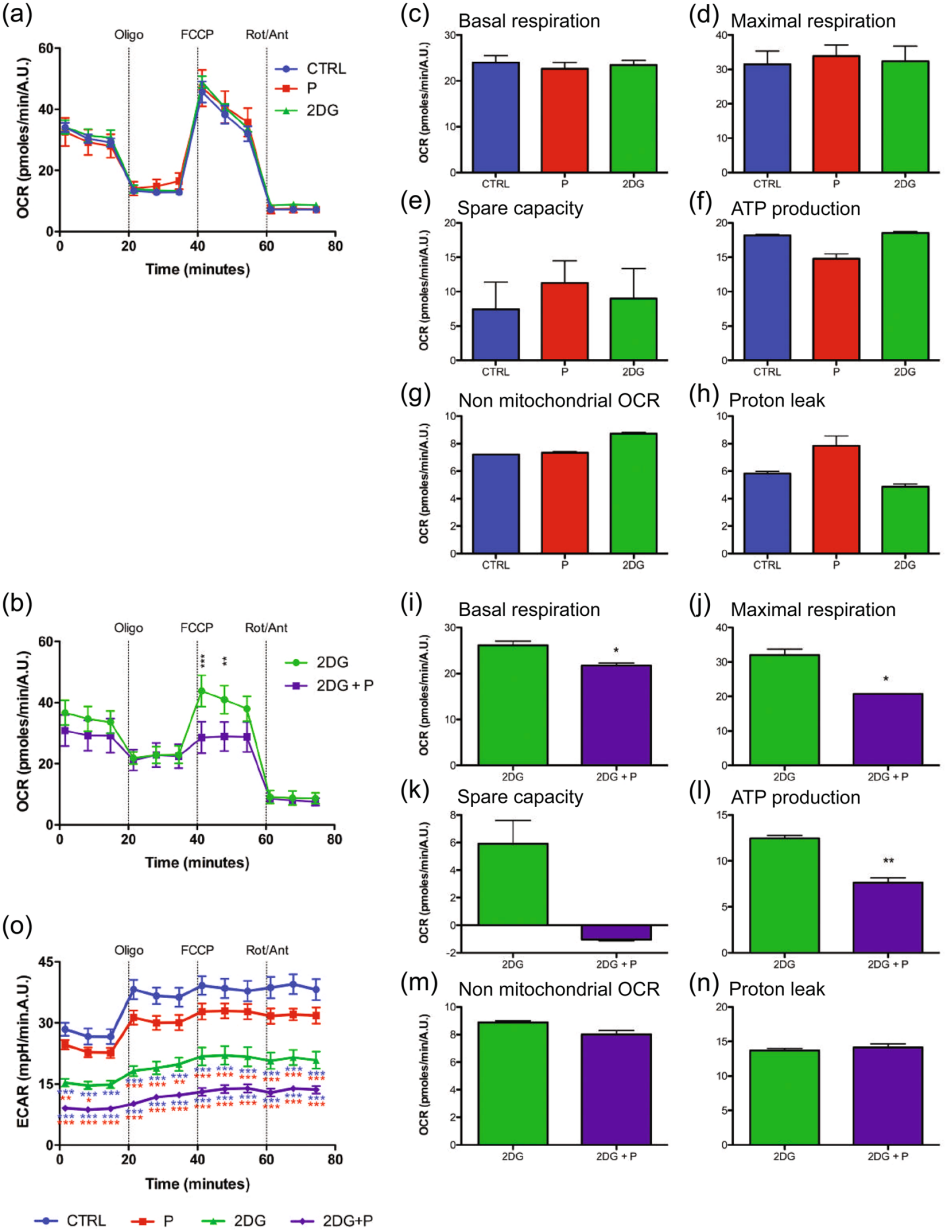
2DG combined with propranolol decreases mitochondrial respiration. PC3 cells were treated with 2DG (2DG) (10 mM), propranolol (P) (100 µM) or both for 24 h. PC3 cells were analyzed for mitochondrial bioenergetics using the Agilent Seahorse XF technology. (**a,b**) Measurement of basal level of oxygen consumption rate (OCR) was followed by sequential injections of oligomycin, FCCP, and rotenone/antimycin A. (**a**) The OCR in control (CTRL), P and 2DG conditions were not significantly different. (**b**) Comparison of 2DG and 2DG+P condition shows a decrease of OCR in the double treated condition. (**c**–**n**) Quantification graph of OCR parameter: (**c,i**) basal and (**d,j**) maximal respirations. Both are reduced in the 2DG+P condition only. (**e,k**) Quantification of spare capacity and (**f,l**) ATP production-related OCR. OCR related to ATP production is significantly decreased in PC3 cells treated with 2DG+P, while spare capacity is not significantly different (p = 0.054). (**g,m**) Quantification of non-mitochondrial OCR and (**h,l**) proton leak. These two parameters are not altered by the double treatment. (**o**) Extracellular acidification rate (ECAR) measured with the Agilent Seahorse XF technology. Measurement of basal level of ECAR was followed by sequential injections of oligomycin, FCCP, and rotenone/antimycin A. The ECAR was not significantly modified in the P condition as compared to the CTRL condition while the ECAR is significantly decreased in the 2DG alone and 2DG+propranolol conditions.

Although accumulation of p62 can be a marker of mitophagy, we did not observe any mitochondria inside double membrane vesicles in PC3 cells with the double treatment (Fig. 5f,h). However, mitochondria exhibited a condensed configuration which was never observed in control cells (see arrows and compare Supplementary Fig. S5a,b). Furthermore, the network of mitochondria, stained in living cells with TMRE, was profoundly altered in PC3 cells exposed to both drugs (Supplementary Fig. S5c,d). The percentage of cells presenting morphological modifications of their mitochondrial network increases progressively with time and reached a maximum after 24 h of treatment (Supplementary Fig. S6). Interestingly, PNT1A control cells were less sensitive than PC3 cells (Supplemental Fig. S7). Modifications of the mitochondrial network suggested an altered mitochondrial function. To assess the effect of treatment on mitochondrial respiration, real-time measurements of the oxygen consumption rate were performed. Propranolol or 2DG treatment alone did not significantly affect mitochondrial activity in PC3 cells (Fig. 7a,c–h). By contrast, the presence of both 2DG and propranolol causes a significant reduction of the basal and maximal respirations as compared to control cultures and to cultures in presence of 2DG or propranolol alone (Fig. 7i,j). In parallel, the ATP production-related respiration was also significantly diminished by the combined treatment (Fig. 7l) while the spare capacity – defined as the difference between maximal and basal respiration – was not significantly decreased (p = 0.054) (Fig. 7k). No modification in non-mitochondrial respiration and in proton leak was observed between 2DG alone and 2DG+ propranolol conditions (Fig. 7m,n). These defects of mitochondrial function were associated with a decreased glycolysis rate as revealed by measurements of the extracellular acidification rate (ECAR) in the various tested conditions (Fig. 7o). These results demonstrated the potent anti-mitochondrial properties of the 2DG and propranolol combination.

### Combined treatment with 2DG and propranolol reduces *in vivo* tumor growth

**Figure 8:**
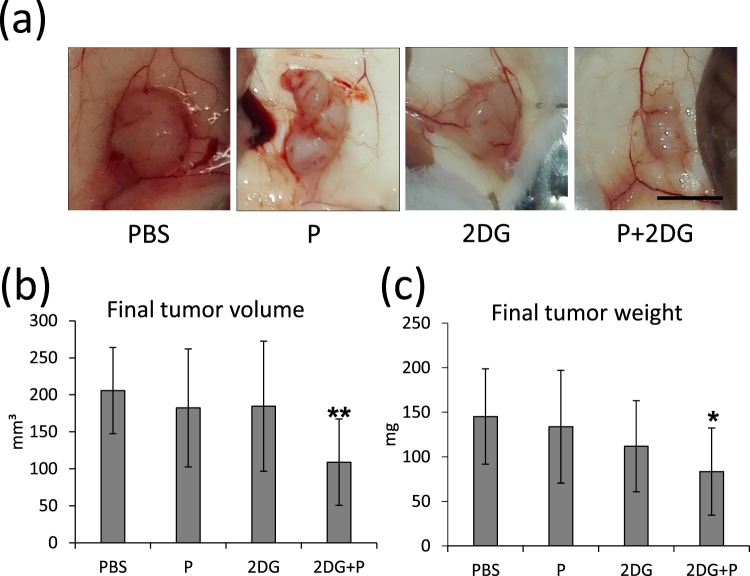
Propranolol and 2DG together inhibit tumor growth *in vivo*. 2 × 10^6^ PC3 cells were injected subcutaneously in each flank of nude mice (n = 20 mice). The tumors were allowed to grow for 11 days and then the animals were separated in four groups (n = 10 tumors in each condition). Each group received a specific treatment: Control (PBS), propranolol (P), 2DG (2DG) and 2DG+P (2DG+P) as described in the “Materials and Methods”. After 16 days, mice were sacrificed. The tumors were macroscopically observed (**a**), measured (**b**) and weighed (**c**). The double treatment induced a significant reduction of tumor size and weight. Bar = 5 mm.

To extend our *in vitro* findings, we examined the effect of the 2DG+ propranolol treatment on *in vivo* tumor growth in mice. Drug dosage was based on literature data and did not induce toxicity. PC3 cells were injected in the flank of NOD SCID mice. After 10 days, mice were randomly separated in four groups and treated daily for 16 days by intraperitoneal injection of vehicle alone (PBS), propranolol (10 mg/kg) alone, 2DG (500 mg/kg) alone or 2DG+propranolol. Only the combined treatment induced a significant decrease of tumor weight and volume at the end of the experiment (Fig. 8) as compared to the control condition (PBS), confirming the interest of using both drugs together.

## Discussion

Retrospective studies on patients treated with propranolol reported a protective effect against various types of cancers, including prostate cancer^15^, and have led to investigation of its anti-tumoral properties^25–27^. Recently, it has been reported that propranolol can be efficiently combined with metabolic inhibitors like rapamycin or metformin to target cancer cells^3,28^. Beside its β-adrenergic receptor blocker activity, propranolol can strongly inhibit the PAP activity of lipins^29–31^. Lipin-1, a member of the lipin family, was reported to be overexpressed in various cancer types and its silencing or inhibition prevents cancer cells proliferation *in vitro* and tumor growth *in vivo*, highlighting the interest of its targeting in anti-cancer therapy^3,32,33^. In a recent study, we observed that phenotypical modifications induced in cancer cells by lipin-1 silencing can be recapitulated by propranolol treatment^3^. Moreover, it is also likely through its ability to inhibit the PAP activity of lipins that propranolol blocks autophagy^5^, a cell survival mechanism induced to resist to stressors like starvation. Hence, we hypothesized that propranolol-treated cancer cells should be sensitized to metabolic stress inducing survival autophagy. This was investigated *in vitro* in low glucose conditions or in presence of the metabolic inhibitor 2DG^21^. As expected, the association of 2DG and propranolol was especially harmful for cancer cells in blocking proliferation and inducing cell death. The blockage by propranolol of the autophagy flux induced by 2DG resulted in a strong accumulation of LC3-II and p62 and in a massive accumulation of autophagic vesicles. Pharmacological aggravation of endoplasmic reticulum (ER)-stress is a principle exploited for cancer therapeutic applications which can be induced by autophagy inhibition^34^. The aggravation of ER-stress observed upon 2DG+ propranolol treatment is likely due to the autophagy blockade mediated by propranolol which would promote cell death by overloading the tumor cells’ adaptation capacity. This is illustrated by the shift of the balance between the antiapoptotic GRP78 and the proapoptotic CHOP towards CHOP which induce cell death. The combined treatment also induced a dramatic disorganization of the mitochondrial network suggesting an imbalance between the fusion and fission states of mitochondria. This equilibrium can be affected by an altered balance between lipid species. Phosphatidic acid and diacylglycerol were reported to regulate the mitochondrial fusion/fission balance^35,36^ and propranolol as well as 2DG can affect the relative concentrations of these two lipid species^37^. As an additional feature, Ca^2+^ can be released from ER stressed by the presence of both 2DG and propranolol and would contribute to mitochondrial fragmentation via the Miro GTPase^38^. By transmission electron microscopy it was also observed that mitochondria in propranolol+2DG-treated cells displayed a condensed morphology. All these morphological alterations were reminiscent of mitochondrial dysfunction. This was confirmed by real-time measurements of the oxygen consumption rate demonstrating a reduction of mitochondrial respiration. In our conditions, insufficient respiration cannot be compensated by increased glycolysis suggesting that lack of intracellular energy could also contribute to the dramatic phenotype observed with the combined treatment. Finally, the significant inhibition of *in vivo* tumor growth in mice treated with 2DG+ propranolol observed here as well as the good tolerance of 2DG and propranolol in clinical trials^20,39^ suggest the possibility to use the combination 2DG+ propranolol in anti-cancer therapies. Our study highlights for the first time the interest to take advantage of the ability of propranolol to inhibit autophagy in cancer. Propranolol appears as a potential alternative to other autophagy modulators which are relatively toxic in noncancerous tissues^40^. More widely, this property of propranolol should be taken into account for the treatment of other pathologies characterized by an excessive autophagy like neurodegenerative diseases.

## Materials and Methods

### Cell lines and reagents

Hoechst H33258 was purchased from Calbiochem (Merck, Overijse, Belgium). Sodium pyruvate, sodium cacodylate, glutamine, glucose, carbonyl cyanide 4-(trifluoromethoxy)-phenylhydrazone (FCCP), oligomycin, rotenone, antimycin A, 2-deoxy-D-glucose (D6134), (±)-propranolol hydrochloride (P0884) were from Sigma (Darmstadt, Germany). Glutaraldehyde was from Bioconnect (The Netherlands). Human prostate adenocarcinoma cells (PC3) were grown in Ham’s F-12K medium (Invitrogen, Merelbeke, Belgium) complemented by 7% Foetal Bovine Serum (FBS) (Lonza, Verviers, Belgium) or cultured in DMEM (Lonza) supplemented with 7% dialyzed FBS and different concentrations of glucose. Human breast adenocarcinoma cells (MDAMB231) and mouse mammary adenocarcinoma cells (4T1) were cultured in DMEM supplemented with 7% FBS. Human immortalized prostatic cells (PNT1A) and prostate carcinoma cells (LnCaP) were grown in RPMI 1640 (Lonza) supplemented with 7% FBS. All media were supplemented with penicilline-streptomycine (100 units/ml each) and Fungizone (250 ng/ml). PC3 cells were authenticated as reported in^3^ (DSMZ, Braunschweig, Germany).

### Western blotting

Cells were solubilised in SDS-PAGE lysis buffer and equal amount of protein were loaded and separated by polyacrylamide gel electrophoresis. After separation, proteins were transferred to a PVDF membrane (NEN Life Science Products), blocked in 3% dry milk in PBS-Tween 20 (0.05%) and probed with the following primary antibodies for 3 hours: rabbit antibodies against lipin-1 (#sc98450), p62/sequestosome 1 (#sc28359) and GRP78 (#sc13968) were from Santa-Cruz Biotechnology (Bioconnect). Rabbit antibody against Erk1/2 (#M5670) was from Sigma, mouse antibody against LC3 (#0260-100) and mouse antibody against CHOP (#2895) were respectively from Nanotools (Merck) and from Cell Signaling (Bioke, Leiden, Netherlands). After the primary antibody exposition, membranes were washed three times with PBS-Tween and incubated 1 h with the diluted secondary antibodies: rabbit anti-mouse IgG (P0260) or swine anti-rabbit IgG (P0217), both conjugated to horseradish peroxidase and purchased from DAKO (Heverlee, Belgium). The blots were then revealed by chemoluminescence using home-made reagents and images were acquired and quantified with ImageQuant™ Las4000© (GE Healthcare Life Sciences). As a loading control, the membranes were re-probed with an antibody against Erk1/2. Erk1/2 was chosen because its expression level is not regulated by cell morphology alterations like cytoskeletal proteins^41^ and unlike GAPDH, another commonly used control, it is not directly involved in cell metabolism.

### Proliferation assay

Cell proliferation was determined by DNA content measurements^42^, as previously described^3,43^. Briefly, cells were seeded in 24-well plates and processed at different time points. The cells were rinsed three times with saline and lyzed in PBS by sonication. In a 96-well plate 100 μl of lysate was mixed 1/1 with a hoechst solution (2 µg/ml hoechst, 4 M NaCl, 0.02 M NaH_2_PO4, pH 7.4). Fluorescence was measured in a spectrofluorometer SpectraMax i3 (Molecular Devices, Sunnyvale, California, USA) (excitation wavelength 355 nm, emission wavelength 460 nm). A DNA standard curve was included in every 96-well plate.

### Mitochondrial network visualization

Mitochondria visualization in living cells was performed using TMRE (tetramethyl rhodamine ethyl ester) (#87917 Sigma) labelling. TMRE is a cationic dye that is rapidly accumulated by mitochondria. 10^5^ cells/well were seeded in 8-wells micro-Slides (#80826, IBIDI, Munich, Germany). At different time points cells were loaded for 20 min at 37 °C with 1 nM TMRE in culture medium and analyzed by epifluorescence microscopy. The acquired images were analyzed with ImageJ software. The network status of the cell was visually characterized as normal if filamentous mitochondria were the only observed mitochondria, intermediate if filamentous mitochondria and puncta mitochondria coexisted and altered if only puncta were present.

### Autophagy analyses

Autophagy flux was investigated using the reporter plasmid pBABE-puro-mCherry-EGFP-LC3B (Addgene) as reported previously^44^. Briefly, PC3 cells were transfected for 16 hours with 1 μg of plasmid per well in 6-well plates. Then, the cells were trypsinized and seeded in 8-wells micro-Slides (IBIDI). After attachment the cells were treated with propranolol, 2DG or cultured in low glucose condition for the indicated times. The images were acquired by epifluorescence microscopy after 24 and 48 hours of treatment and the ratio between early (red and green) and late autophagosomes (red only) was quantified with the ImageJ software. The analyses were performed on stacked images of several focal planes.

### Cell survival and apoptosis

Cell survival and apoptosis were evaluated by FACS analysis following manufacturer instructions of the Annexin V-FITC Apoptosis Detection kit (Sigma). Flow cytometry was performed on a FACS-Canto II double LASER flow cytometer (UV, 488 nm, 633 nm) (BD Biosciences, Franklin Lakes, New Jersey) and data were analyzed using FACSDiva Software (BD Biosciences). The cells negative for both Annexin-V and PI are considered as “live cells” while cells positive for either one or both markers are considered “dead cells”.

### Extracellular Flux Analysis for determination of respiratory capacities

10^4^ cells/well were seeded in Seahorse XFp mini-plates (Agilent, Santa Clara, California) and allowed to attach overnight. After 24 hours drug treatment, cells were kept in unbuffered serum-free XF assay medium (Agilent) supplemented with pyruvate (1 mM), glutamine (2 mM), glucose (10 mM), pH7.4 at 37 °C and ambient CO2 for one hour before the assay. During the assay, cells were successively stressed with oligomycin (1 µM), FCCP (0,5 µM) and a rotenone/antimycine A (0.5 µM each) mix. FCCP concentration was chosen according to^45^. The readout of the assay was the oxygen consumption rate (pmoles/min). Results were normalized according to the cell number evaluated by Hoechst (2 µg/ml) incorporation after cold methanol/acetone (4:1) fixation.

### Transmission electron microscopy (TEM) analysis

At the end of the 24 hour drugs treatments, cells were fixed for 1 h in a 0.1 M sodium cacodylate buffer (pH 7.4) containing 2.5% glutaraldehyde (v/v) and post-fixed for 30 min with 2% (w/v) osmium tetroxide in the same buffer. Samples were then dehydrated at room temperature through an increasing ethanol series (70%, 96% and 100%) and embedded in Epon for 48 h at 60 °C. Ultrathin sections (70 nm thick) were obtained by using an ultramicrotome (Reichert Ultracut E Leica, Wetzlar, Germany), equipped with a diamond knife (Diatome, Hatfield, Pennsylvania). The sections were mounted on copper grids coated with collodion and contrasted with uranyl acetate and lead citrate for 15 min each. The ultrathin sections were observed under a JEM-1400 transmission electron microscope (Jeol, Croissy Sur Seine, France) at 80 kV and micrographs were taken with an 11 MegaPixel bottom-mounted TEM camera system (Quemesa, Olympus, Münster, Germany).

### Animal experiments

Experimental protocol was approved by the ethical committee of the University of Liège (approval document n°1701) according to the European Communities Council Directive. NOD SCID male mice were bred and housed in pathogen-free condition. All procedures were performed in accordance with the Federation of European Laboratory Animal Science Associations (FELASA) guidelines. A volume of 200 μl of serum-free medium containing 2 × 10^6^ PC3 cells was inoculated into both flanks of mice (6–8 weeks old, n = 20). After 10 days, tumors had reached an average volume of 50 mm^3^. Mice were then treated daily with intra-peritoneal injections (100 µL) of PBS (control group), of propranolol (10 mg/kg), of 2DG (500 mg/kg), or of both drugs. After 16 days of treatment mice were sacrificed by cervical dislocation and decapitated. After resection tumor weight were measured and tumor sizes were assessed by measuring the length and width of tumors, and the volume was determined by using the following formula: (length) × (width)^2^ × 0.4. The data are presented as means ± s.d.

### Statistical analyses

Statistical analyses were performed using Mann-Whiney U test or ANOVA test followed by Tukey-Kramer post-test (GraphPad Prism software, GraphPad Sofware, La Jolla, CA, USA) with p ≤ 0.05 considered as significant. In the figures data are expressed as mean ± standard deviations of biological triplicates, annotations are as follow: not significant (ns), p < 0.05 (*)p < 0.01 (**)p < 0.001 (***).

### Data availability Statement

All data generated or analysed during this study are included in this published article and its Supplementary Information files.

## Electronic supplementary material

supplementary figures

uncropped blots
