# Supplementary material for: Propranolol sensitizes prostate cancer cells to glucose metabolism inhibition and prevents cancer progression
Source: Sci Rep. 2018 May 4;8:7050. doi: 10.1038/s41598-018-25340-9 (PMC5935740; doi:10.1038/s41598-018-25340-9)
Supplement: Supplementary file 1 — supplementary figures [file 41598_2018_25340_MOESM1_ESM.pdf]

Supplementary Information

Propranolol sensitizes prostate cancer cells to glucose metabolism inhibition and prevents cancer progression.

Authors: Laura Brohée[1], Olivier Peulen[2], Betty Nusgens[1], Vincent Castronovo[2], Marc Thiry[3], Alain C. Colige[1] and Christophe F. Deroanne[1]

Supplementary Figure Legends

Supplementary Figure S1: No accumulation of early autophagosomes is observed in low glucose PC3 cells

Autophagy flux was investigated in PC3 cells transfected with an expression vector for LC3-eGFP-mCherry as described in "Materials and Methods" and in previously published results [3]. PC3 cells were cultured with 1mM or 7mM glucose for 24 or 48h. (**a**) Representative fluorescent microscopy photographs are shown (scale bars = 10 µm). (**b**) Percentages of early/late autophagosomes after 24 and 48h of treatment as determined in at least 40 cells per condition (mean ± s.d.). No accumulation of autophagosomes is observed in 1mM glucose condition.

Supplementary Figure S2: No accumulation of early autophagosomes is observed in 2DG-treated PC3 cells

Autophagy flux was investigated in PC3 cells transfected with an expression vector for LC3-eGFP-mCherry as described in "Materials and Methods" and in previously published results [3]. PC3 cells were treated either with or without 10mM 2DG for 24 or 48h. The graph represents the percentages of early/late autophagosomes after 24 and 48h of treatment as determined in at least 40 cells per condition (mean ± s.d.). No accumulation of autophagosomes is observed in 2DG-treated PC3.

Supplementary Figure S3: Propranolol and 2DG combined treatment results in massive accumulation of LC3II in MDAMB231 cells.

MDAMB231 were left untreated or treated with 2DG (10 mM), propranolol (P) (100 µM) or both together. Autophagy was investigated by immuno-blotting against LC3-II and p62 and normalized to Erk1/2. LC3-II and p62 are significantly increased in 2DG+P condition compared to single treatment and control condition.

Supplementary Figure S4: Propranolol and 2DG added together induce vesicles accumulation on the Golgi apparatus

PC3 cells were collected after 24h of treatment (control or 2DG+P) and prepared for electron microscopy analysis as detailed in "Materials and Methods". (**a**, **b**) Golgi apparatus of control cells shows a typical conformation (see arrows in **a**), while vesicles accumulate on the trans face of the Golgi in the 2DG+P treated condition (see arrows in **b**) (scale bars = 1 µm).

Supplementary Figure S5: 2DG combined with propranolol induces mitochondrial stress and network disorganization

Cells were left untreated (C) or treated with 2DG (10 mM), propranolol (P) (100 µM) or both together for 24h. (**a**, **b**) Electron microscopy comparison of control (**a**) or 2DG+P (**b**) treated cells. Mitochondria in control cultures (see arrows in **a**) have a clear matrix with numerous cristae perpendicular to their long axis. The mitochondrial matrix of 2DG+P-treated cells (see arrows in **b**) appears condensed with a disorganization of cristae (scale bars = 1µm). (**c**, **d**) Mitochondria membrane network was analyzed after TMRE loading as described in "Materials and Methods" showing mitochondrial network in treated cells. A fragmented mitochondrial network is observed in 2DG+P-treated cells only (scale bars = 10 µm). (**d**) The graph represents the percentages of cell with either altered (red), intermediate (orange) or normal (grey) mitochondrial network analyzed as detailed in "Materials and Methods". The numbers of evaluated cells were respectively: 172 (C), 192(P), 180 (2DG) and 1227 (2DG+P) cells.

Supplementary Figure S6: Time course of mitochondrial network alteration in PC3 cells treated with propranolol and 2DG added together

PC3 cells were treated with 2DG (10 mM) and propranolol (P) (100 μM) together for the indicate time. Mitochondria membrane networks were stained and analyzed after TMRE loading as described in the "Materials and Methods". The graph represents the percentages of cell with either altered (red), intermediate (orange) or normal (grey). The mitochondrial network of 2DG+P-treated cells is increasingly altered over time. The numbers of evaluated cells were respectively: 224(2h), 354 (4h), 319 (8h), 1227 (24h) and 210 (48h) cells.

Supplementary Figure S7: Propranolol and 2DG combination affects the aggressive PC3 prostate cancer cell line but is less effective on the PNT1A control cells

PC3 cells and PNT1A cells were treated with 2DG (10 mM) and propranolol (P) (100 μM) together, for the indicated times. Mitochondria membrane network was analyzed after TMRE loading as described in "Materials and Methods". The graph represents the percentages of cells with either altered (red), intermediate (orange) or normal (grey) mitochondrial network analyzed as detailed in "Materials and Methods". A fragmented mitochondrial network is observed in 72% 2DG+P-treated PC3 cells but only in 36% of PNT1A cells. The numbers of evaluated cells were 658 for PNT1A and 1227 PC3 cells.

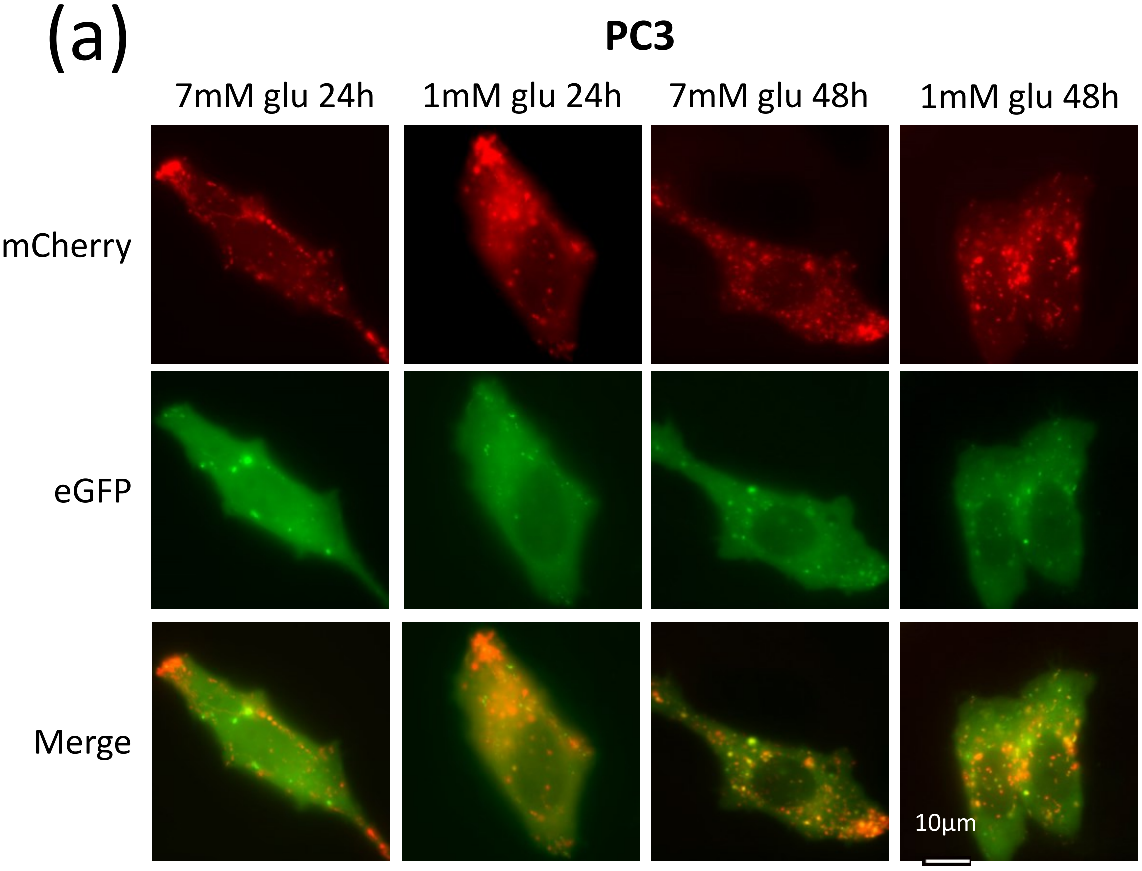

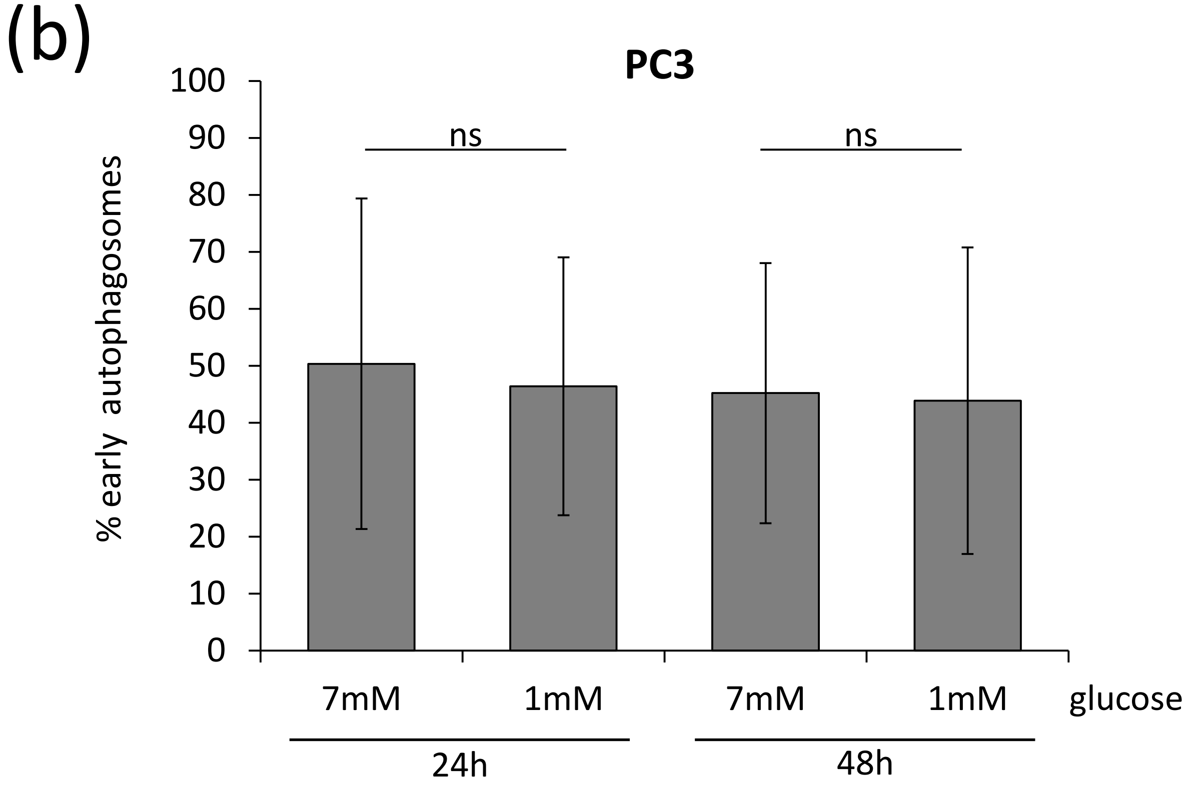

Supplementary Figure S1

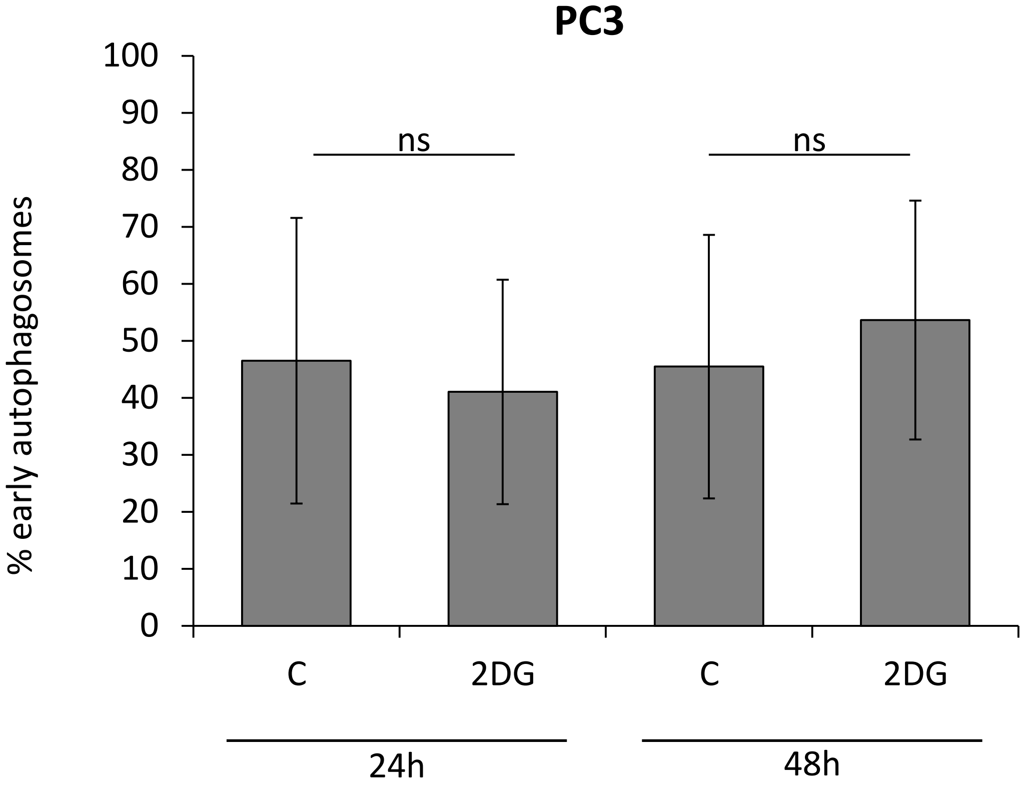

Supplementary Figure S2

MDAMB231

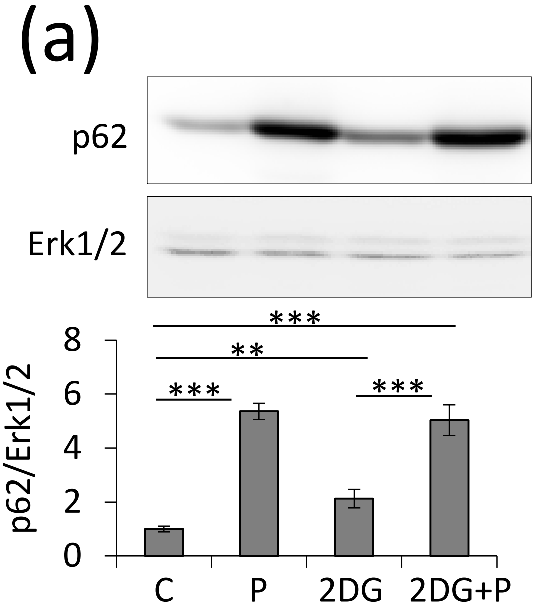

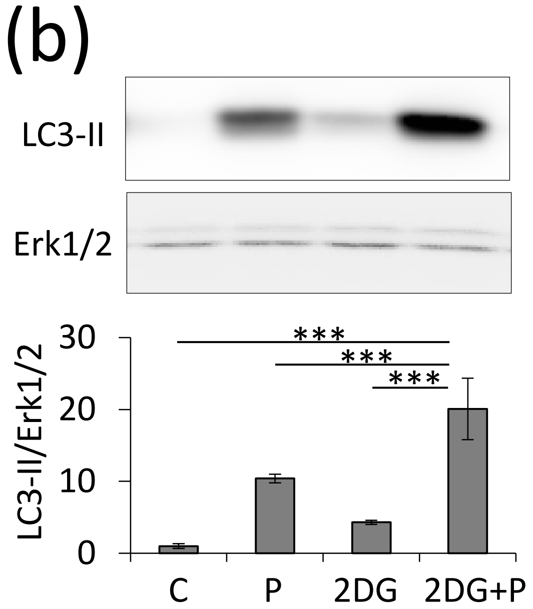

Supplementary Figure S3

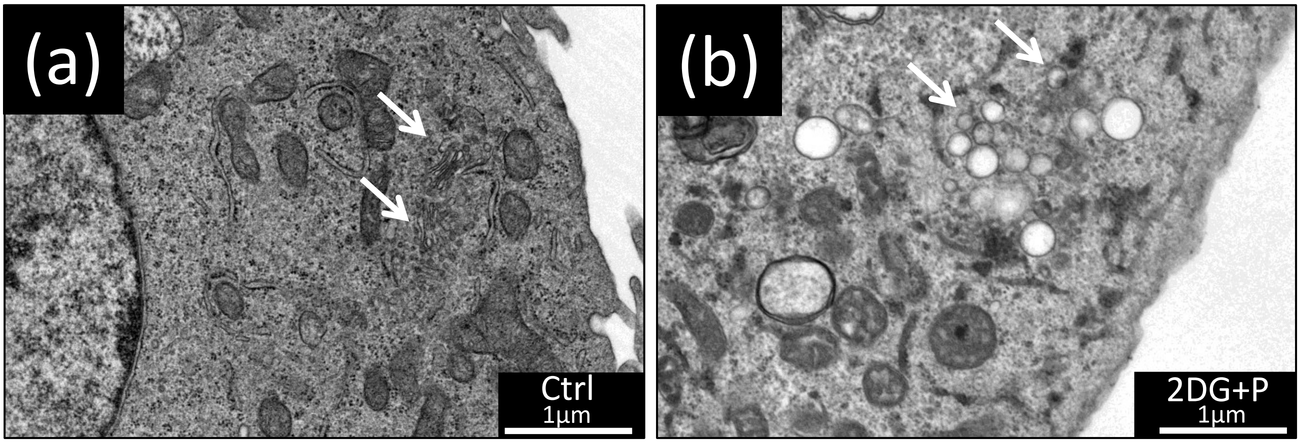

Supplementary Figure S4

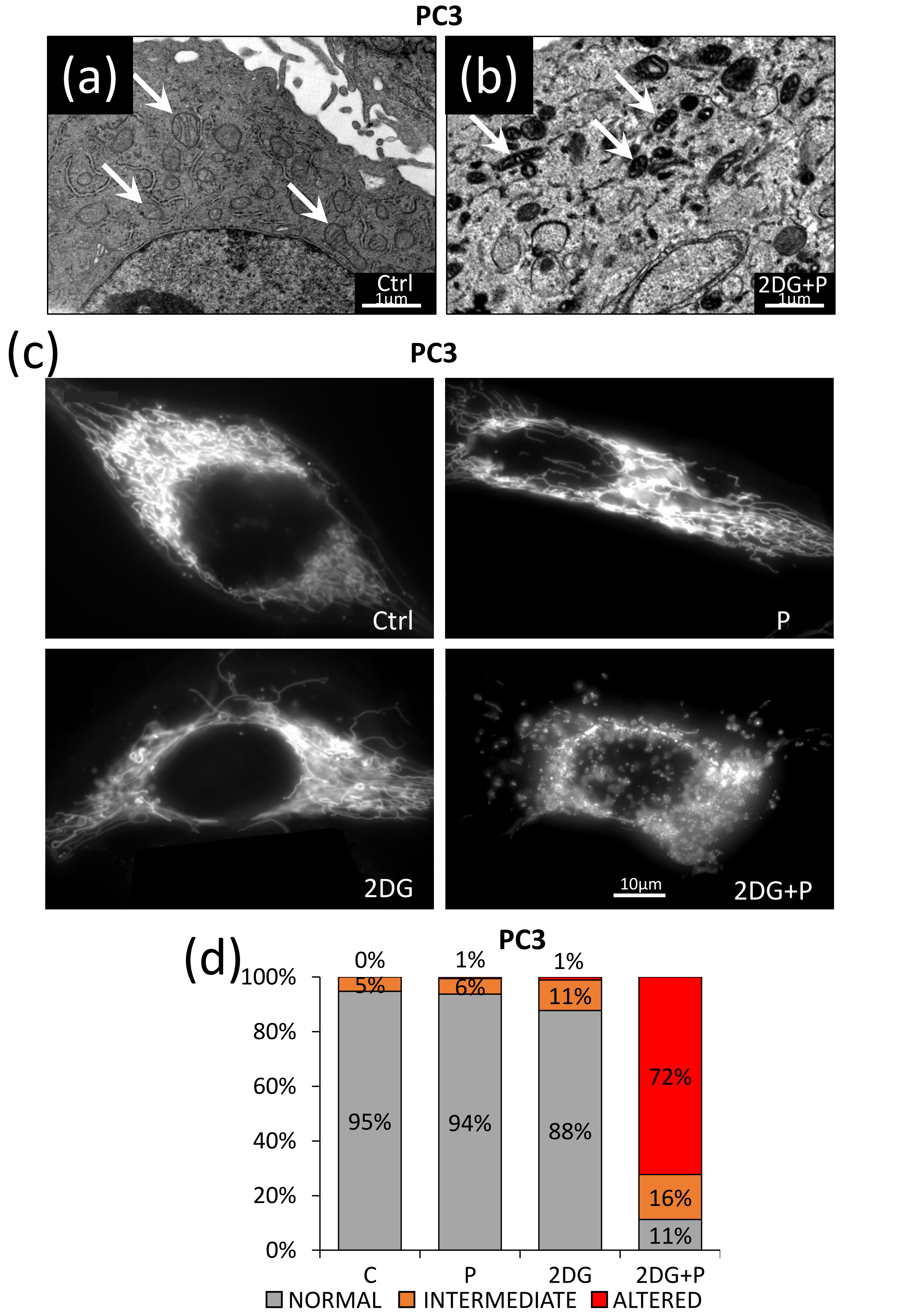

Supplementary Figure S5

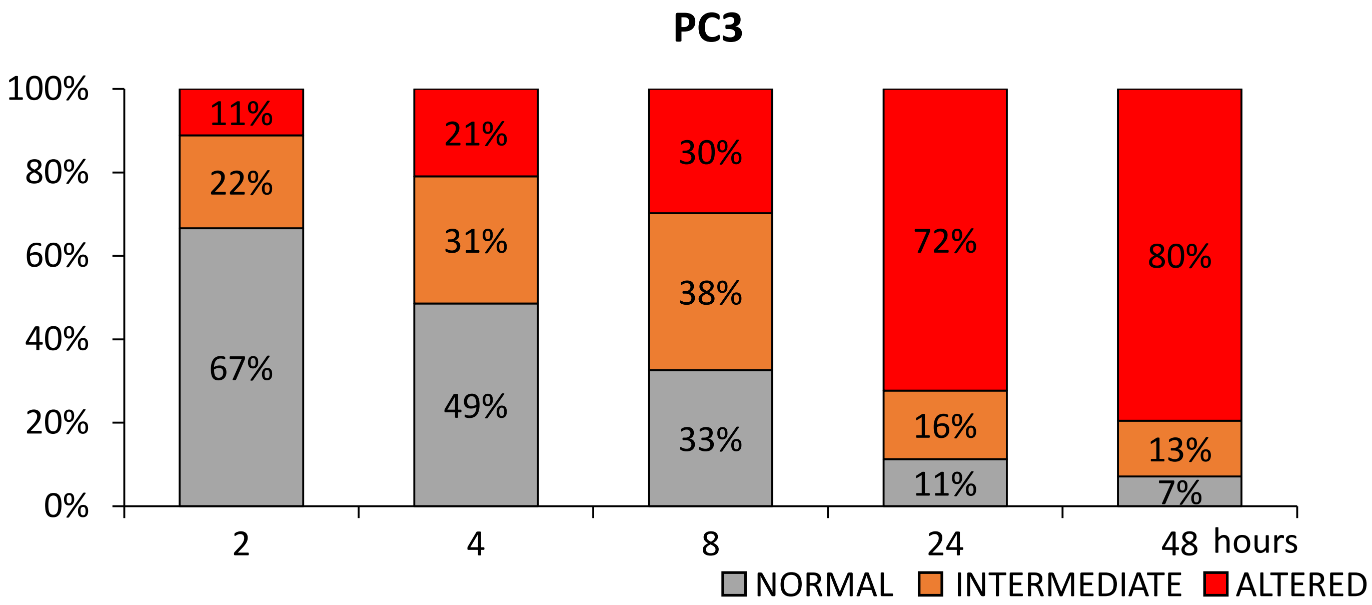

Supplementary Figure S6

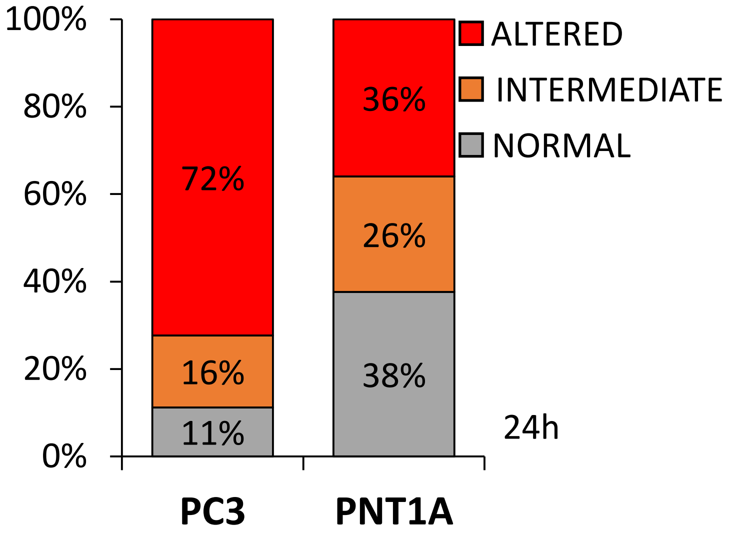

Supplementary Figure S7
