# Supplementary material for: Propranolol sensitizes prostate cancer cells to glucose metabolism inhibition and prevents cancer progression
Source: Sci Rep. 2018 May 4;8:7050. doi: 10.1038/s41598-018-25340-9 (PMC5935740; doi:10.1038/s41598-018-25340-9)

Supplementary Information

Propranolol sensitizes prostate cancer cells to glucose metabolism inhibition and prevents cancer progression.

Authors: Laura Brohée[1], Olivier Peulen[2], Betty Nusgens[1], Vincent Castronovo[2], Marc Thiry[3], Alain C. Colige[1] and Christophe F. Deroanne[1]

Uncropped blots

Figure 1(a) upper left LC3

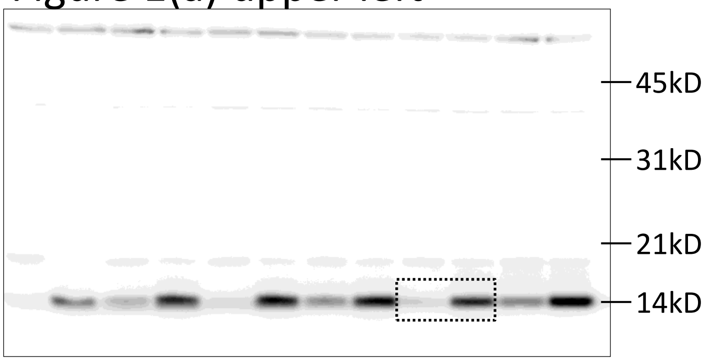

Figure 1(a) upper left Erk1/2

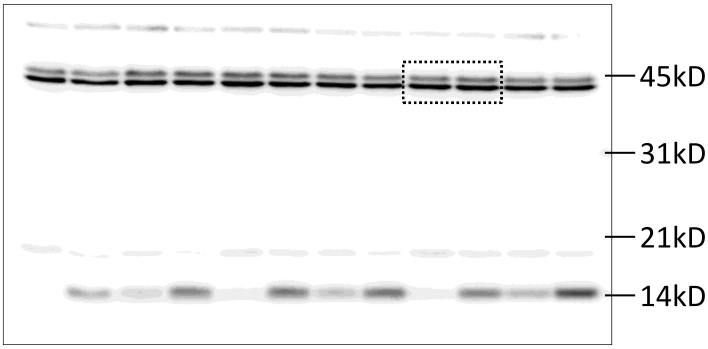

Figure 1(a) lower left p62

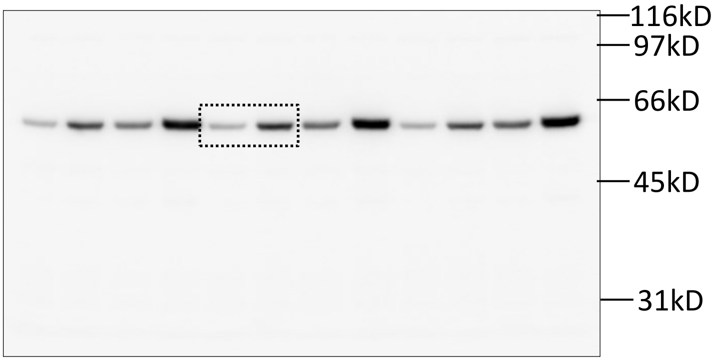

Figure 1(a) lower left Erk1/2

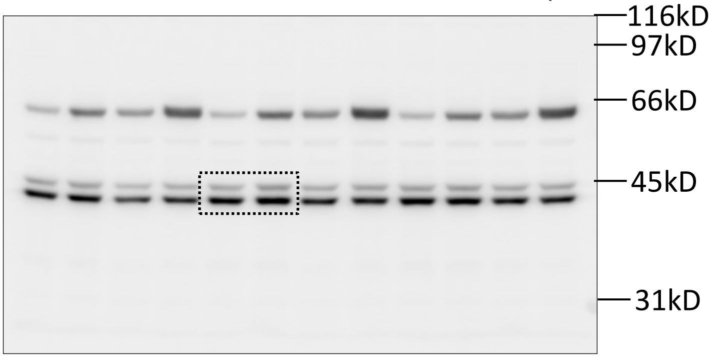

Figure 1(a) upper right LC3

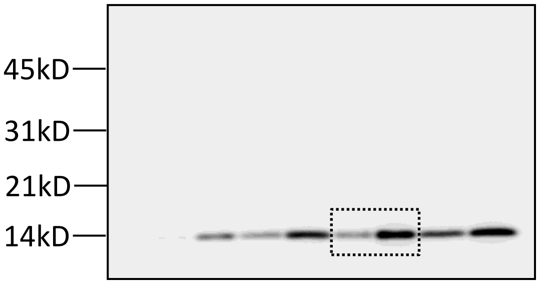

Figure 1(a) upper right Erk1/2

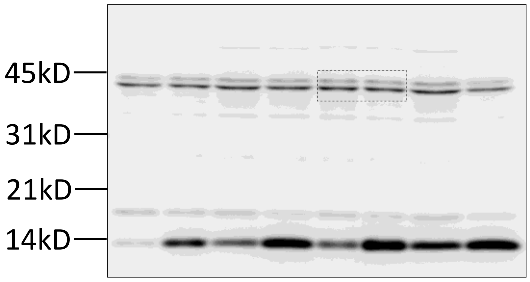

Figure 1(a) lower right p62

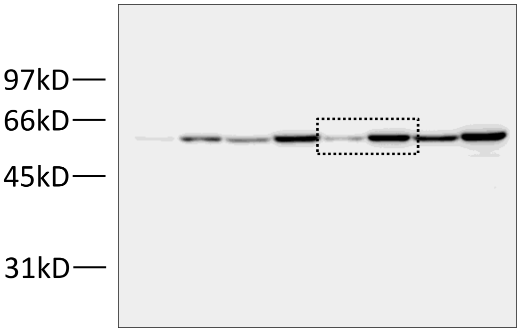

Figure 1(a) lower right Erk1/2

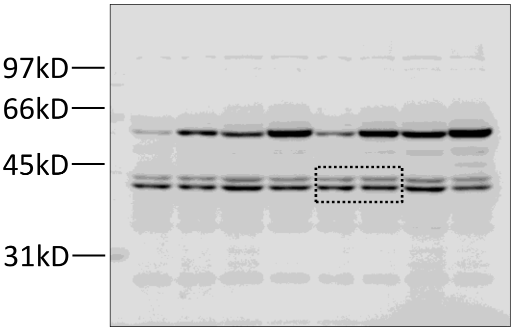

Figure 2(a) upper LC3

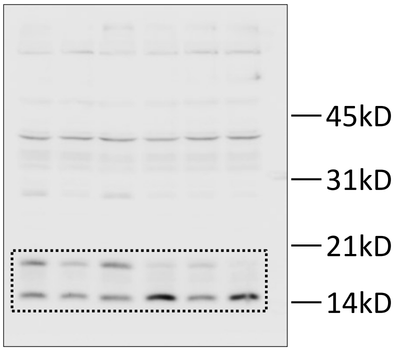

Figure 2(a) upper Erk1/2

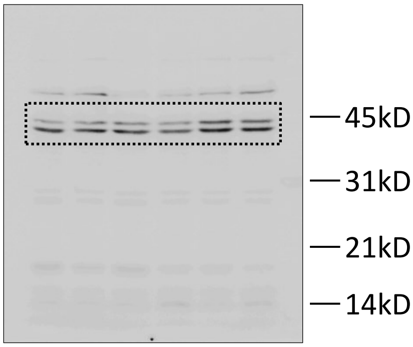

Figure 2(a) lower p62

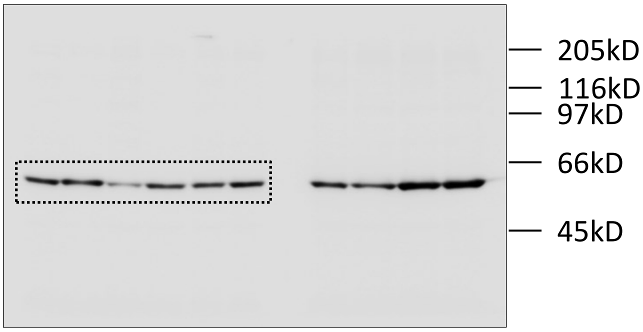

Figure 2(a) lower Erk1/2

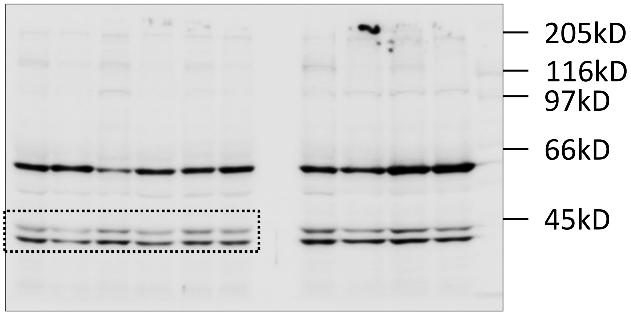

Figure 2(b) upper LC3

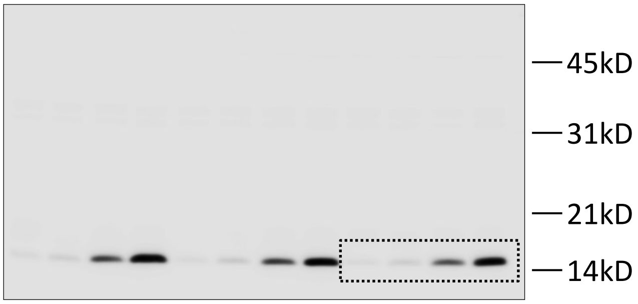

Figure 2(b) upper Erk1/2

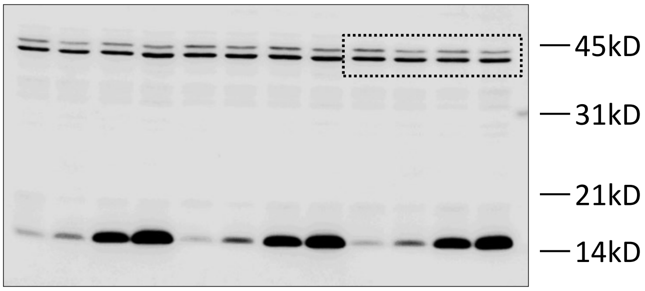

Figure 2(b) lower p62

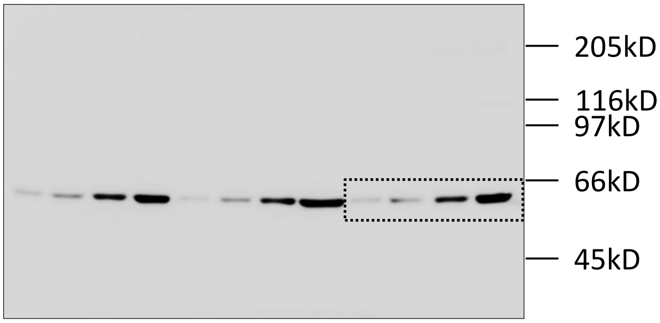

Figure 2(b) lower Erk1/2

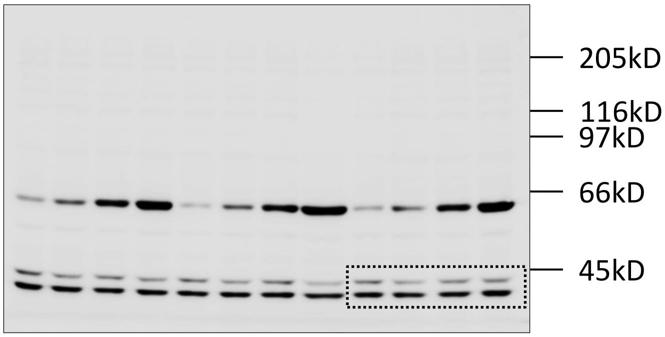

Figure 3(a) upper left

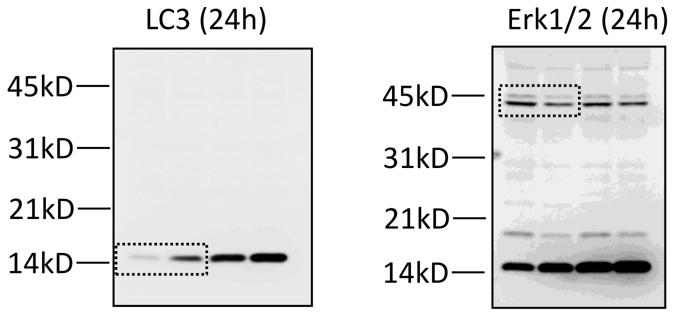

Figure 3(a) upper right

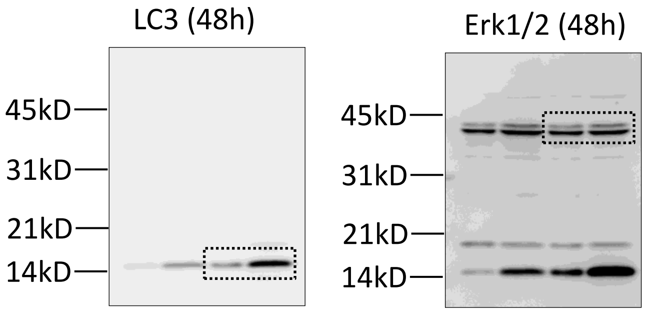

Figure 3(a) lower left

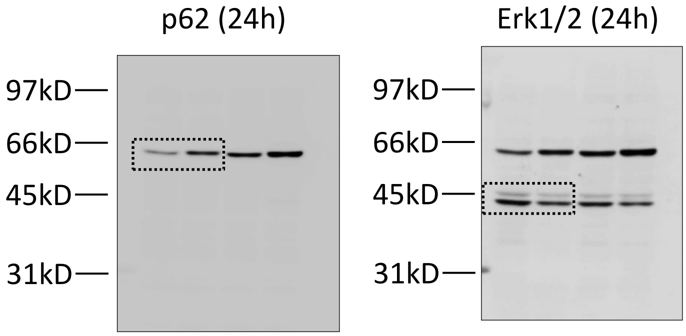

Figure 3(a) lower right

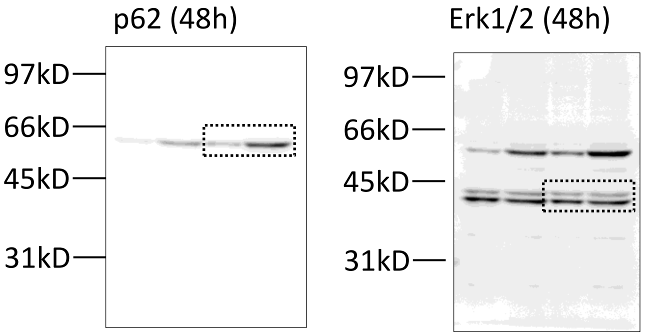

Figure 3(b) upper

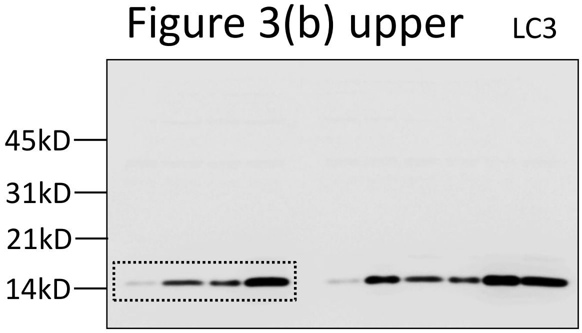

Figure 3(b) upper

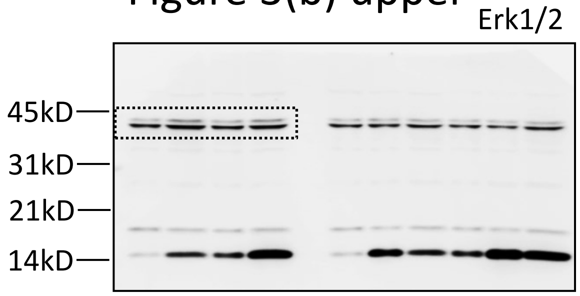

Figure 3(b) lower

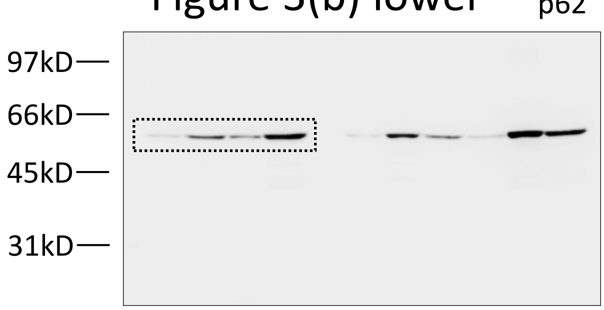

Figure 3(b) lower

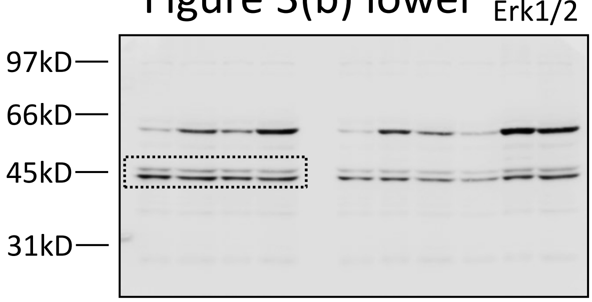

Figure 5(a) upper

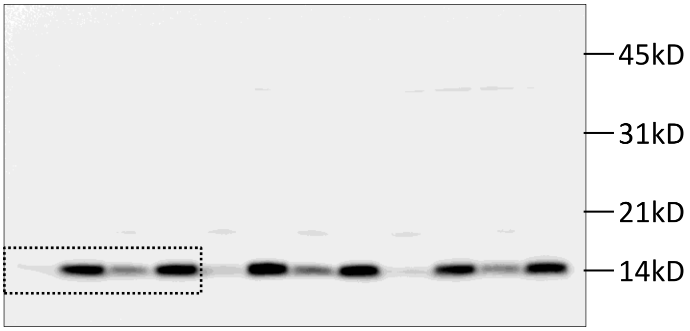

Figure 5(a) upper

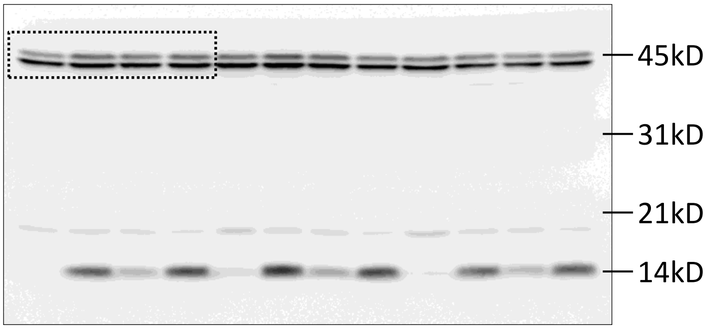

Figure 5(a) lower

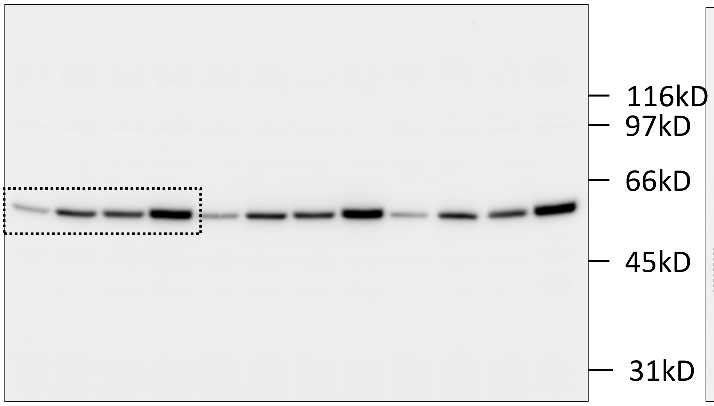

Figure 5(a) lower

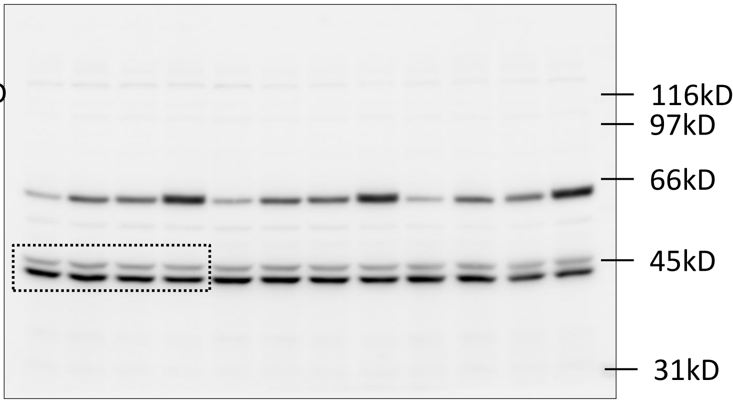

Figure 5(b) upper

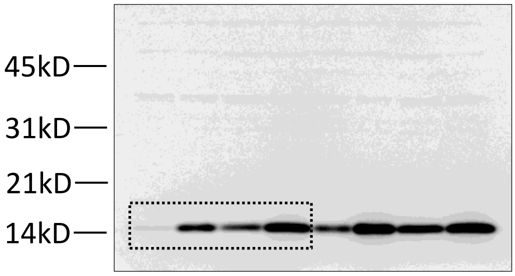

Figure 5(b) upper

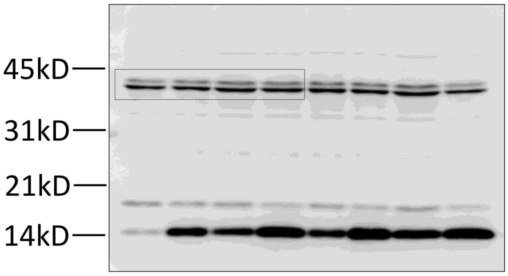

Figure 5(b) lower

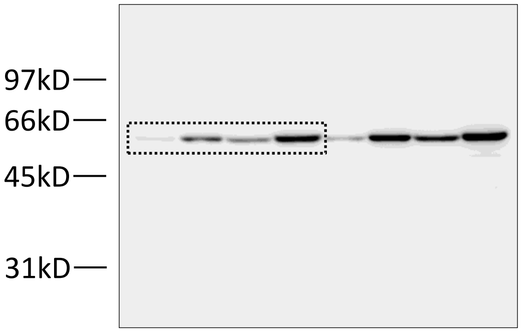

Figure 5(b) lower

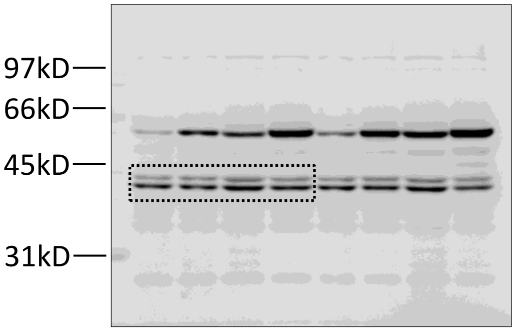

Figure 6(c) CHOP

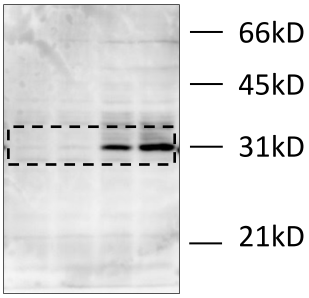

Figure 6(c) Erk1/2

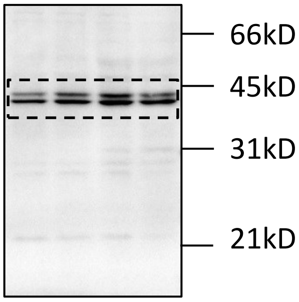

Figure 6(d) GRP78

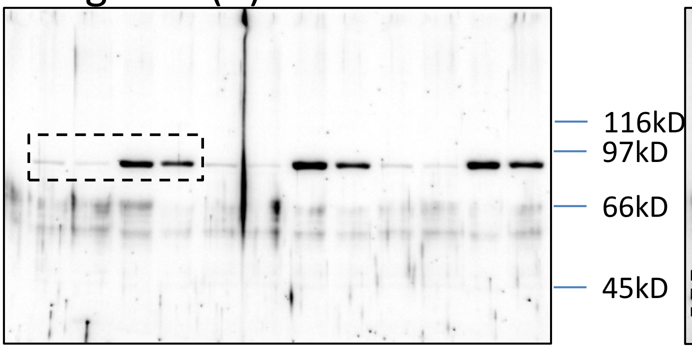

Figure 6(d) Erk1/2

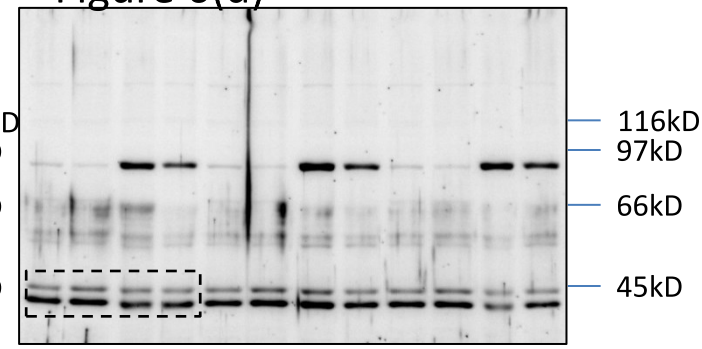

Figure 6(e) CHOP

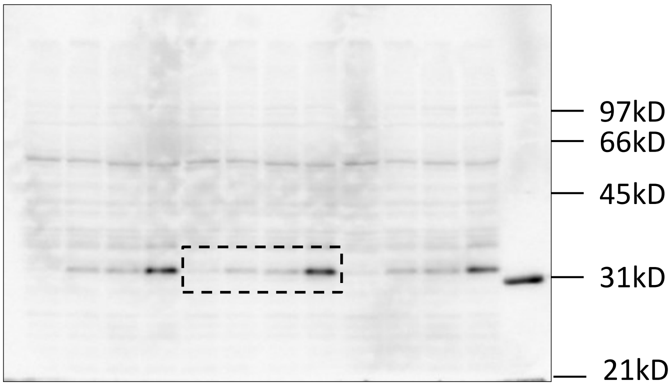

Figure 6(e) Erk1/2

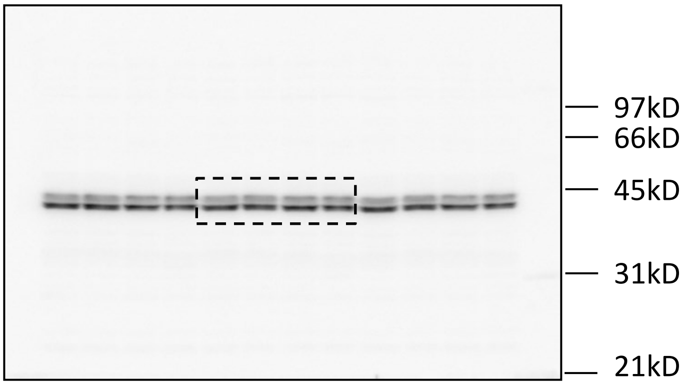

Figure 6(f) GRP78

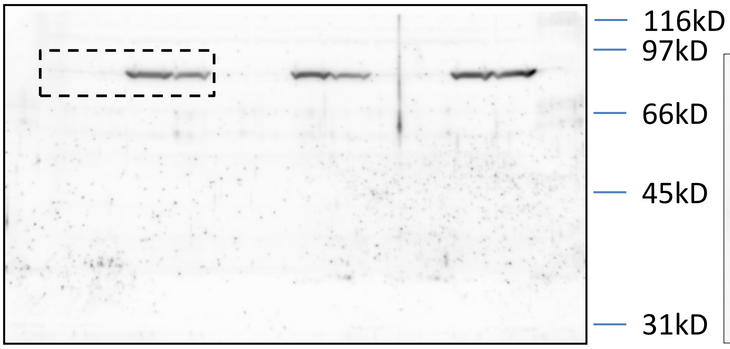

Figure 6(f) Erk1/2

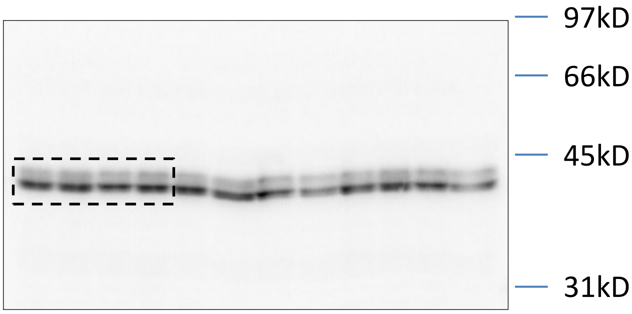

Supplement: Supplementary file 2 — uncropped blots [file 41598_2018_25340_MOESM2_ESM.pdf]
